# Supplementary material for: Non-Markovian intracellular transport with sub-diffusion and run-length dependent detachment rate
Source: PLoS One. 2018 Nov 26;13(11):e0207436. doi: 10.1371/journal.pone.0207436 (PMC6261056; doi:10.1371/journal.pone.0207436)
Supplement: S1 File — Fig A, A single motor stepping in a part of experimental trajectory. The distance L travelled by a cargo is shown as a function of time. Fig B, The distribution of cargo position increments d r = r(t + Δ) − r(t) in experimental trajectories calculated at time interval Δ = 0.1 s. The probability density function (PDF) is shown as a function of the displacement (d r). Fig C, Time averaged mean displacement (TAMEAN) for experimental trajectories of the lipid droplets as a function of time interval D together with linear trend D (dashed line). Fig D, (a) Time averaged mean squared displacement (TAMSD) for experimental trajectories of the lipid droplets as a function of time interval D, (b) TAMSD for trajectories generated by the non-Markovian rate model and (c) by the Markovian rate model. Power-law scaling trends are shown with ∼ Δ0.7 (dashed-dotted line) and Δ2 (dashed line). Fig E, The average velocity of a cargo manually estimated from the experimental trajectory. Parts of the trajectory with different average velocity are marked with capital letters. The figure legend provides with the values of the average velocities (v) corresponding to different parts of trajectory (units μm/s). Fig F, The distribution of average cargo velocities in the experimental trajectory shown in Fig E. Peaks in the distribution correspond to different velocities of the cargo at parts of the trajectory indicated with capital letters next to the arrows. Fig G, Distribution of average cargo velocities in two experimental trajectories (a) and (b). Peaks in the distributions correspond to different cargo velocities. Fig H, Distribution of average cargo velocities in trajectories generated by the non-Markovian rate model (a) and (b) and the Markovian rate model (c) and (d). Fig I, (a) Distance L(t)=x2(t)+y2(t) travelled by cargoes as a function of time for the non-Markovian rate model with the FGE. Parameters are given in the text. (b) Time averaged variances (TAVAR) for single trajectories as [file pone.0207436.s001.pdf]

Supporting information to "Non-Markovian intracellular transport with sub-diffusion and run-length dependent detachment rate" by Nickolay Korabel, Thomas A. Waigh, Sergei Fedotov and Viki J. Allan.

Figure A shows a part of a single experimental trajectory[1]. Single motor protein steps of length 8 nm typical for single dynein motor are observed in the cargo trajectories. Some smaller 5 nm steps were also found. The analysis of the part of the trajectory was done manually and therefore was not applied to other trajectories. Instead, we calculate the distribution of cargo position increments $d\mathbf{r}(\Delta) = \mathbf{r}(t+\Delta) - \mathbf{r}(t)$ (Fig. B). The distribution of cargo position increments calculated using 8 experimental trajectories reveals motor steps of length 8 nm and 18 nm. With such an analysis we avoid manual segmentation of each trajectory to determine the size of motor protein steps.

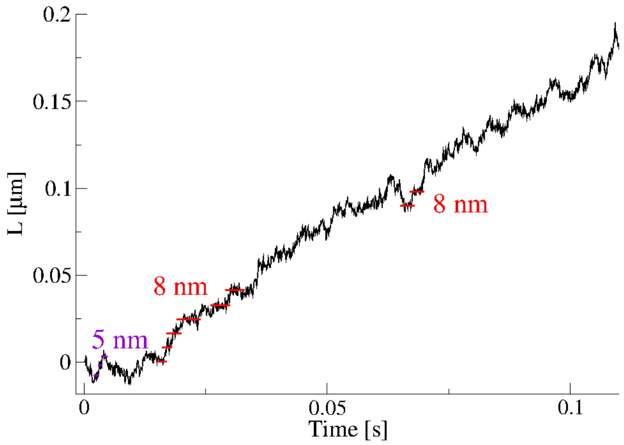

Figure A. A single motor stepping in a part of experimental trajectory[1]. The distance L travelled by a cargo is shown as a function of time. Steps of 5 and 8 μm are highlighted.

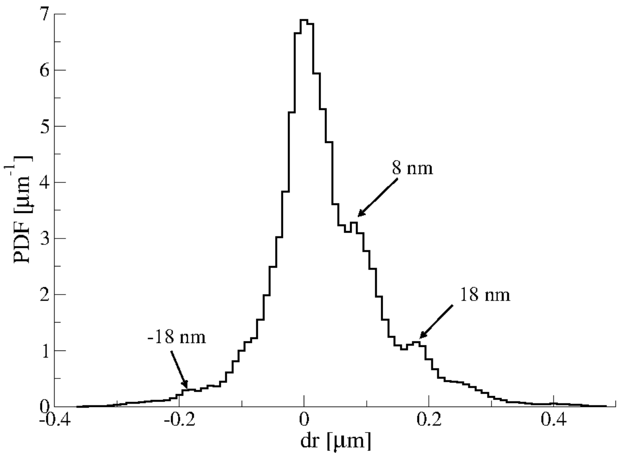

Figure B. The distribution of cargo position increments $d\mathbf{r} = \mathbf{r}(t+\Delta) - \mathbf{r}(t)$ in experimental trajectories calculated at time interval $\Delta = 0.1$ s[1]. The probability density function (PDF) is shown as a function of the displacement ($d\mathbf{r}$).

Next we calculate the time averaged mean displacement (TAMD) of experimental trajectories (Fig. C). Due to processive nature of trajectories, the TAMD grows linearly with time interval Δ. The time averaged mean squared misplacement (TAMSD) is therefore ballistic (Δ^2) (Fig. D).

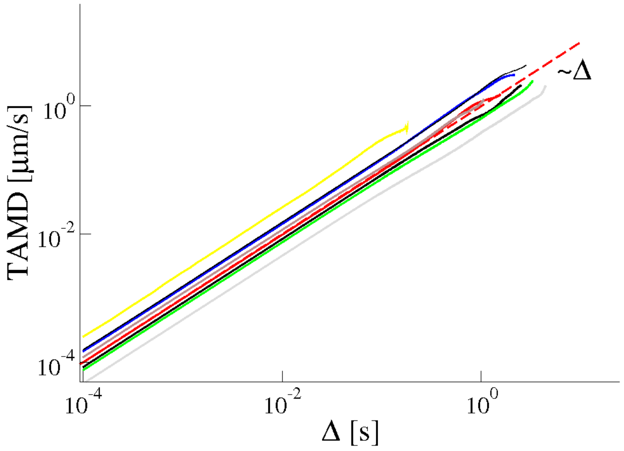

Figure C. Time averaged mean displacement (TAMD) for experimental trajectories of the lipid droplets as a function of time interval Δ together with linear trend Δ (dashed line).

The average velocity of a cargo manually estimated in a sample experimental trajectory is shown in Fig. E. The figure clearly demonstrates that the cargo is moving with different average velocities in different parts of the trajectory. The value of average cargo velocity (given in the figure in the legend) changes from 0 to 2 $\mu m/s$. To avoid manual estimation of the average cargo velocities, we calculated the distribution of average cargo velocity in the experimental trajectories (see Fig. F and Methods for details). Distinct peaks in the distribution corresponds to different velocities of the cargo in different parts of trajectory. Comparing Fig. E and Fig. F, we find that the distribution provides an accurate estimation of average velocities of the cargo without manual segmentation of trajectories.

An example of distributions of average cargo velocities in two experimental trajectories is shown in Fig. G. Again, peaks in the distribution correspond to different cargo velocities confirming the method. More examples of distributions of average cargo velocities in trajectories generated by the non-Markovian rate model is shown in Fig. H.

Non-Markovian rate model with fractional Langevin equation

To show that our results are robust, we implemented the fractional Langevin equation (FLE) into our non-Markovian rate model. The FLE was simulated using the method and parameters described in Ref.[2]:

$$\eta_0 \dot{x} = F_x - \sum_{i=1}^{N} \hat{k}_i (x - x_i) + \sqrt{2\eta_0 k_B T}\,\xi_{0,x}(t), \tag{1}$$

$$\eta_i \dot{x}_i = \hat{k}_i (x - x_i) + \sqrt{2\eta_i k_B T}\,\xi_{i,x}(t), \quad i = 1, \ldots, N,$$

$$\eta_0 \dot{y} = F_y - \sum_{i=1}^{N} \hat{k}_i (y - y_i) + \sqrt{2\eta_0 k_B T}\,\xi_{0,y}(t),$$

$$\eta_i \dot{y}_i = \hat{k}_i (y - y_i) + \sqrt{2\eta_i k_B T}\,\xi_{i,y}(t), \quad i = 1, \ldots, N,$$

where x, y are coordinates of the cargo, η_0, η_i are viscous friction constants x_i, y_i are auxiliary variables, F_x, F_y are components of force vector $\mathbf{F}(t) = \{F_x, F_y\}$ exerted on the cargo by the motor and $\xi_{0,x}(t)$, $\xi_{0,y}(t)$, $\xi_{i,x}$, $\xi_{i,y}$ are uncorrelated white Gaussian noises of unit intensity.

Following Ref.[2] we set

$$\hat{k}_i = \nu_0 \eta_{eff} \frac{b^{1-\alpha} - 1}{b^{(i-1)\alpha}[b^{N(1-\alpha)} - 1]}. \tag{2}$$

The motor dynamics is governed by the non-Markovian rate model (see the main text) with parameters $\mu = 1.4$, $\tau_d = 0.6 - 0.06$ s, $\mathbf{T}_a = 1 - 100$ s^{-1}. The following parameter values were used in Eq. (4): $k_B T = 4.1 \cdot 10^{-21}$ N·m, $N = 8$, $\alpha = 0.7$, $b = 10$, $\nu_0 = 0.1$, $\tilde{\eta}_{eff} = \eta_{eff}/\eta_0 = 10^4$, $D_0 = 0.01 - 0.72$ μm^2/s. For a spherical cargo with the radius 300 nm moving in water with viscosity $\zeta = 10^{-3}$ Pa·s one estimates the friction coefficient $\eta_0 \equiv 5.65 \cdot 10^{-9}$ N·s/m. Friction coefficients η_i are given by $\eta_i = \hat{k}/\nu_i$ where $\nu_i = \nu_0/b^{i-1}$.

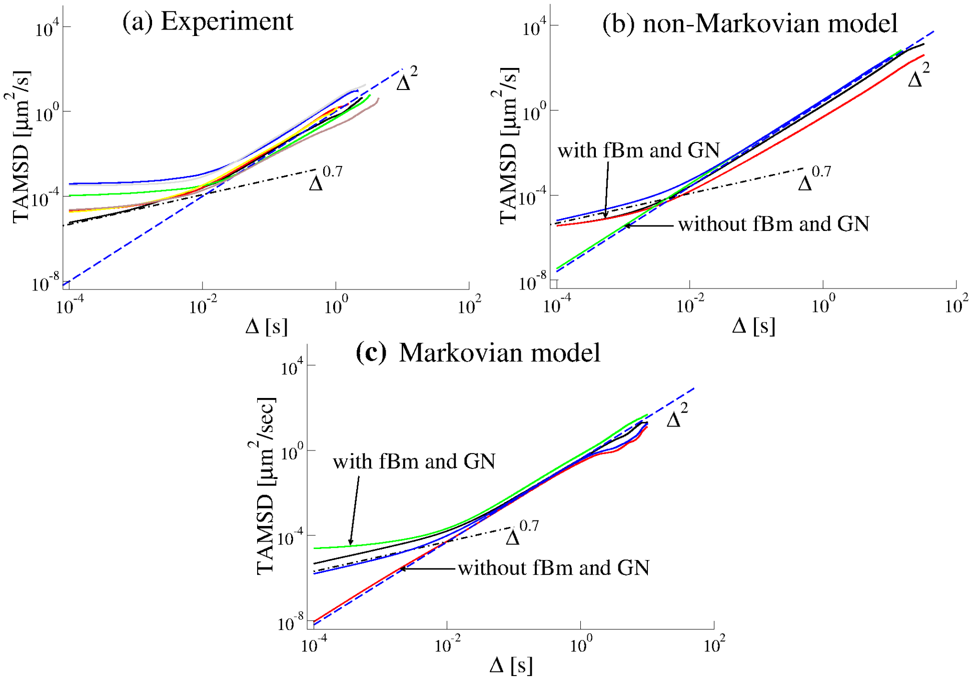

Figure D. (a) Time averaged mean squared displacement (TAMSD) for experimental trajectories of the lipid droplets as a function of time interval Δ, (b) TAMSD for trajectories generated by the non-Markovian rate model and (c) by the Markovian rate model. Power-law scaling trends are shown with $\sim \Delta^{0.7}$ (dashed-dotted line) and Δ^2 (dashed line).

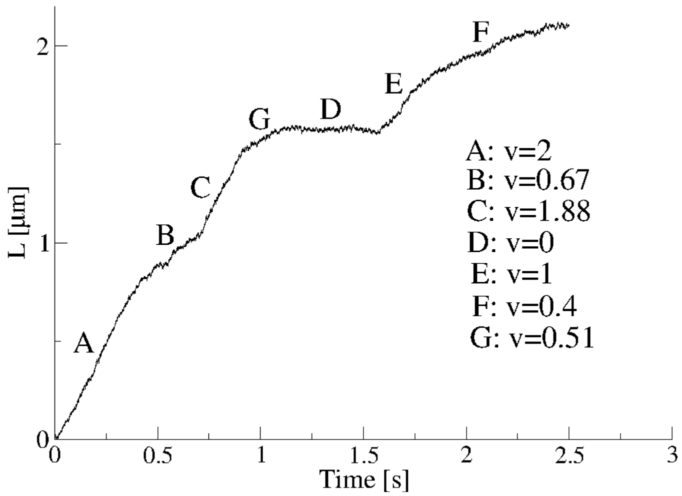

Figure E. The average velocity of a cargo manually estimated from the experimental trajectory. Parts of the trajectory with different average velocity are marked with capital letters. The figure legend provides with the values of the average velocities (v) corresponding to different parts of trajectory (units μm/s).

For the non-Markovian rate model we have used the same parameter values as described in the main text. In particular, for the fractional Gaussian noise we have used $H = 0.35$.

The results of simulations are shown in Fig. I for single trajectories of cargo-motor complex we plotted the distance travelled as the function of time and the time averaged variances. The results are similar to those obtained for the non-Markovian rate model with the external fGn: the short time behaviour is sub-diffusive with the exponent $\alpha = 0.7$, while on the longer time

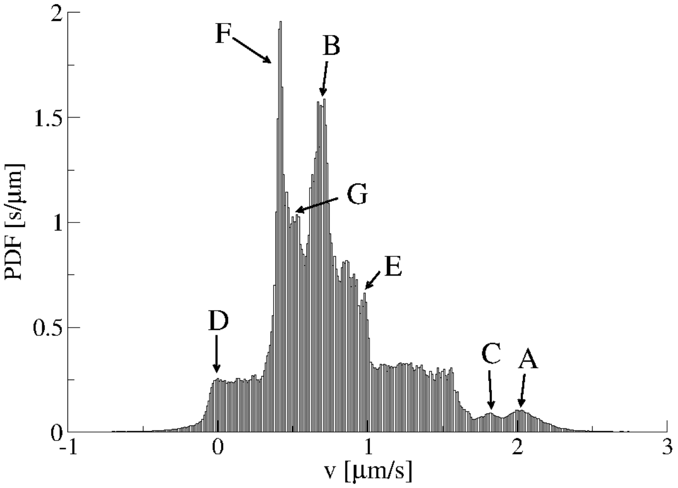

Figure F. The distribution of average cargo velocities in the experimental trajectory shown in Fig. E. Peaks in the distribution correspond to different velocities of the cargo at parts of the trajectory indicated with capital letters next to the arrows .

scale the behaviour is super-diffusive with the exponent $3-\mu = 1.6$ due to the non-Markovian detachment rate. These results confirm that the super-diffusive behaviour in the non-Markovian rate model is not effected by the nature of sub-diffusive motion. Based on this indications, we expect other results to be robust.

References

1. Kenwright, D. A., Harrison, A. W., Waigh, T. A., Woodman, P. G., and Allan, V. J. First-passage-probability analysis of active transport in live cells. Phys. Rev. E **86**, 031910 (2012).
2. Goychuk I, Kharchenko VO, Metzler R. (2014) How molecular motors work in the crowded environment of living cells: coexistence and efficiency of normal and anomalous transport. PLoS ONE 9: e91700.

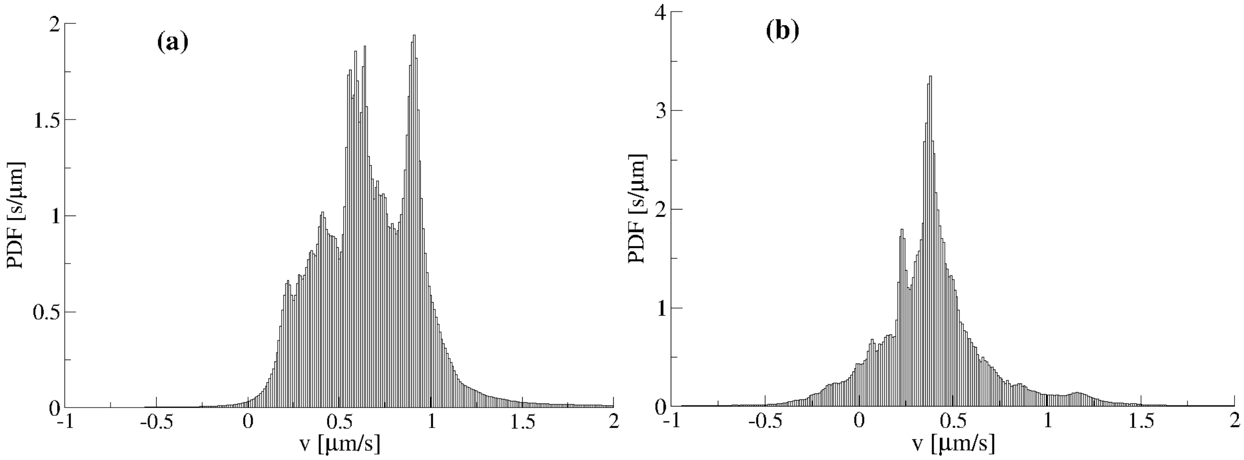

Figure G. Distribution of average cargo velocities in two experimental trajectories (a) and (b). Peaks in the distributions correspond to different cargo velocities.

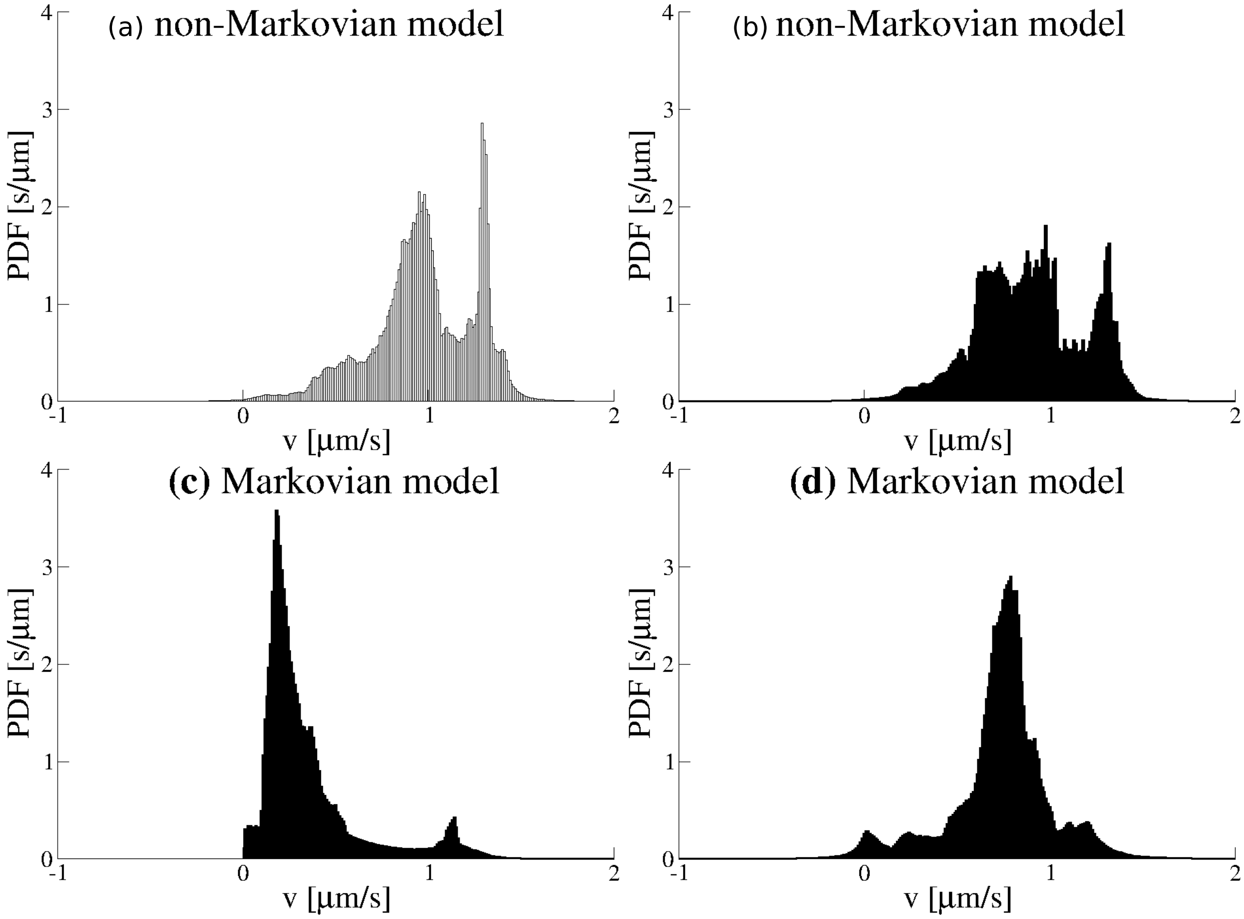

Figure H. (a), (b) distributions of average cargo velocities in trajectories generated by the non-Markovian rate model and (c), (d) distributions of average cargo velocities in trajectories generated by the Markovian rate model.

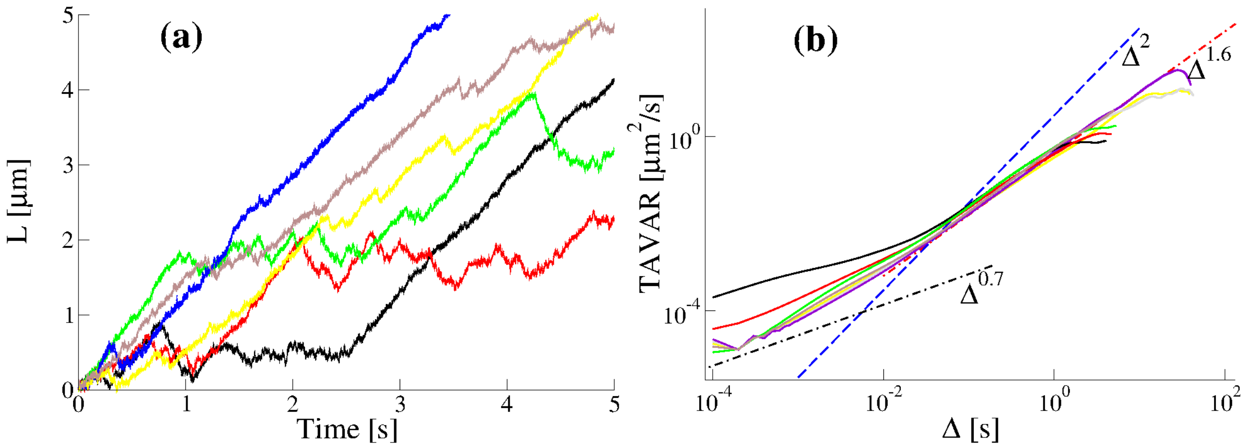

Figure I. (a) Distance $L(t) = \sqrt{x^2(t) + y^2(t)}$ travelled by cargoes as a function of time for the non-Markovian rate model with the FGE. Parameters are given in the text. (b) Time averaged variances (TAVAR) for single trajectories as a function of time interval Δ. Trajectories were of 32 s duration. TAVARs for several longer trajectories of 72 s duration are also shown.
